# Tailored Vascular Approach in Robot‐Assisted Nephroureterectomy for a Horseshoe Kidney: A Case Report

**DOI:** 10.1002/iju5.70212

**Published:** 2026-06-11

**Authors:** Takanari Kambe, Yosuke Shimizu, Hajime Yoshida, Koken Hayashi, Shotaro Hatano, Tomoaki Okada, Kenji Mitsumori, Hiroyuki Onishi

**Affiliations:** ^1^ Department of Urology Osaka Red Cross Hospital Osaka Japan; ^2^ Department of Surgery Osaka Red Cross Hospital Osaka Japan

**Keywords:** horseshoe kidney, indocyanine green fluorescence imaging, radical nephroureterectomy, robot‐assisted surgery, urothelial carcinoma

## Abstract

**Introduction:**

A horseshoe kidney is a rare congenital anomaly with complex vascular variations that increase surgical difficulty. Few reports have specifically addressed the vascular approaches in robot‐assisted surgery.

**Case Presentation:**

A man in his 70s was diagnosed with right ureteral urothelial carcinoma in a horseshoe kidney. Preoperative imaging revealed a short renal artery arising posterior to the isthmus, suggesting difficulty with conventional surgical approaches. Surgery was initiated in the supine position using a medial approach, similar to the medial‐to‐lateral dissection used for sigmoid colon mobilization, with cranial dissection along the aorta to secure the vessel and isthmus, followed by repositioning and standard robot‐assisted nephroureterectomy. Intraoperative indocyanine green fluorescence was used to guide safe isthmus division. The procedure was completed safely without any perioperative complications.

**Conclusion:**

A tailored approach based on detailed preoperative vascular assessment may facilitate safe and effective robot‐assisted surgery in patients with horseshoe kidney and complex vascular anatomy.

## Keynote Message

Preoperative imaging allowed us to identify a renal artery that was difficult to control using the conventional approach and to adjust the surgical procedure. This tailored strategy enabled safe robotic surgery without complications. Individualized planning may improve the outcomes in complex kidney surgeries.

**Abbreviations**
CT: computed tomography
ICG: indocyanine green

## 1 Introduction

Horseshoe kidney is a congenital renal anomaly observed in approximately 0.15%–0.25% of the population [1, 2]. It is frequently associated with vascular and urinary tract anomalies [3], increasing surgical complexity. Although robot‐assisted surgery is increasingly used as a minimally invasive technique, its application in horseshoe kidney remains limited [4, 5, 6, 7]. We report a case of robot‐assisted nephroureterectomy for upper tract urothelial carcinoma in a patient with a horseshoe kidney, using a unique positioning and surgical approach.

## 2 Case Presentation

A man in his 70s presented to a referring hospital with right flank pain and gross hematuria. CT revealed a right upper ureteral stone and a horseshoe kidney. During transurethral lithotripsy, a papillary tumor was identified at the site of stone impaction. A biopsy suggested urothelial carcinoma, and the patient was referred to our hospital.

**FIGURE 1:**
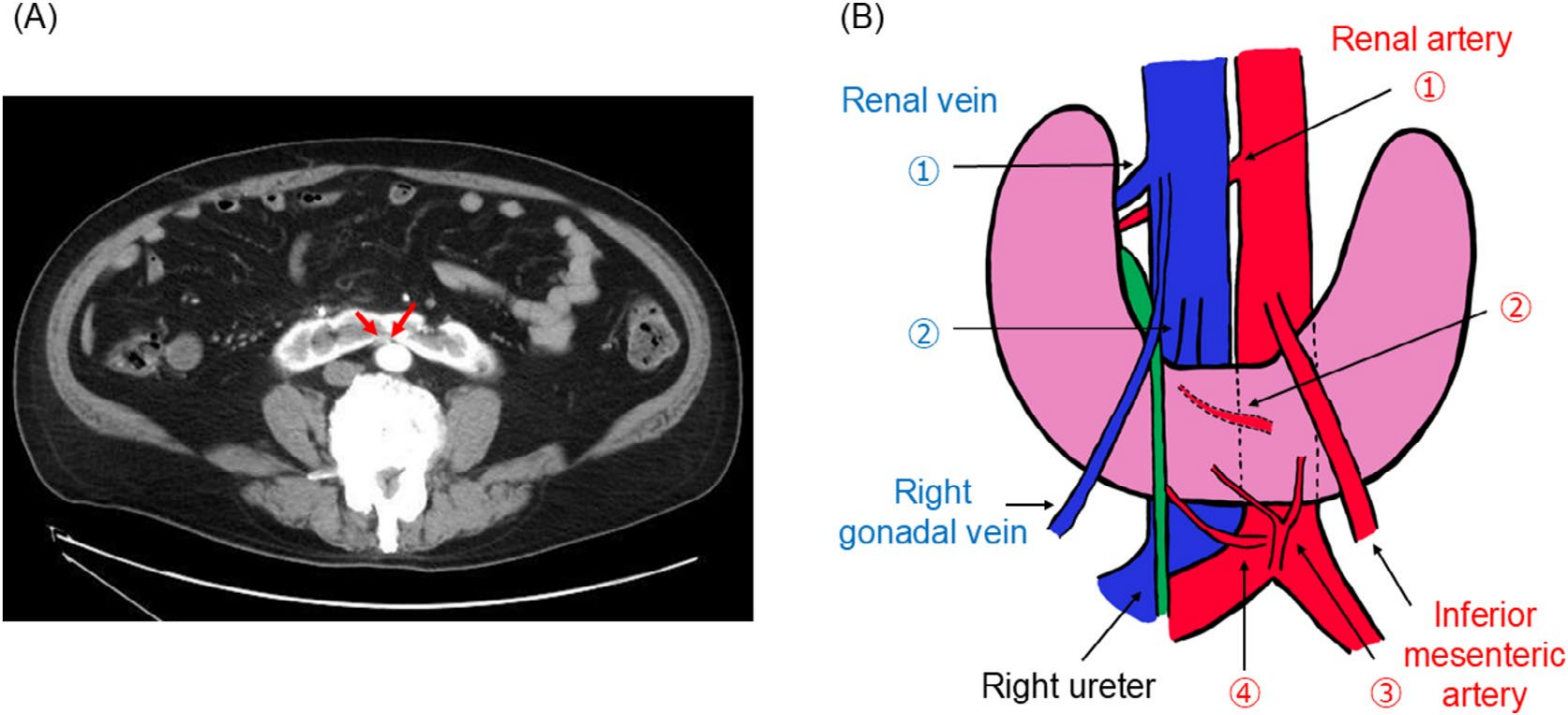
Preoperative assessment of the vascular anatomy. (A) Contrast‐enhanced CT showing a renal artery posterior to the isthmus. (B) Schematic illustration of the anatomical structures of the right renal artery, vein, and ureter in the present case. No. 2 denotes the renal artery of interest.

Retrograde pyelography showed a 3 cm filling defect in the right lower ureter, and selective urine cytology showed a positive result. The patient was diagnosed with a right ureteral urothelial carcinoma (cT1N0M0). Contrast‐enhanced CT demonstrated fusion of the isthmus at L3–L4 level (30 mm wide, 15 mm thick). Four renal arteries and two renal veins supplied the right kidney. The ureter coursed ventral to the isthmus without other urinary tract anomalies. One renal artery originated from the aorta posterior to the isthmus and entered the dorsal aspect with a short length (Figure 1A,B).

**FIGURE 2:**
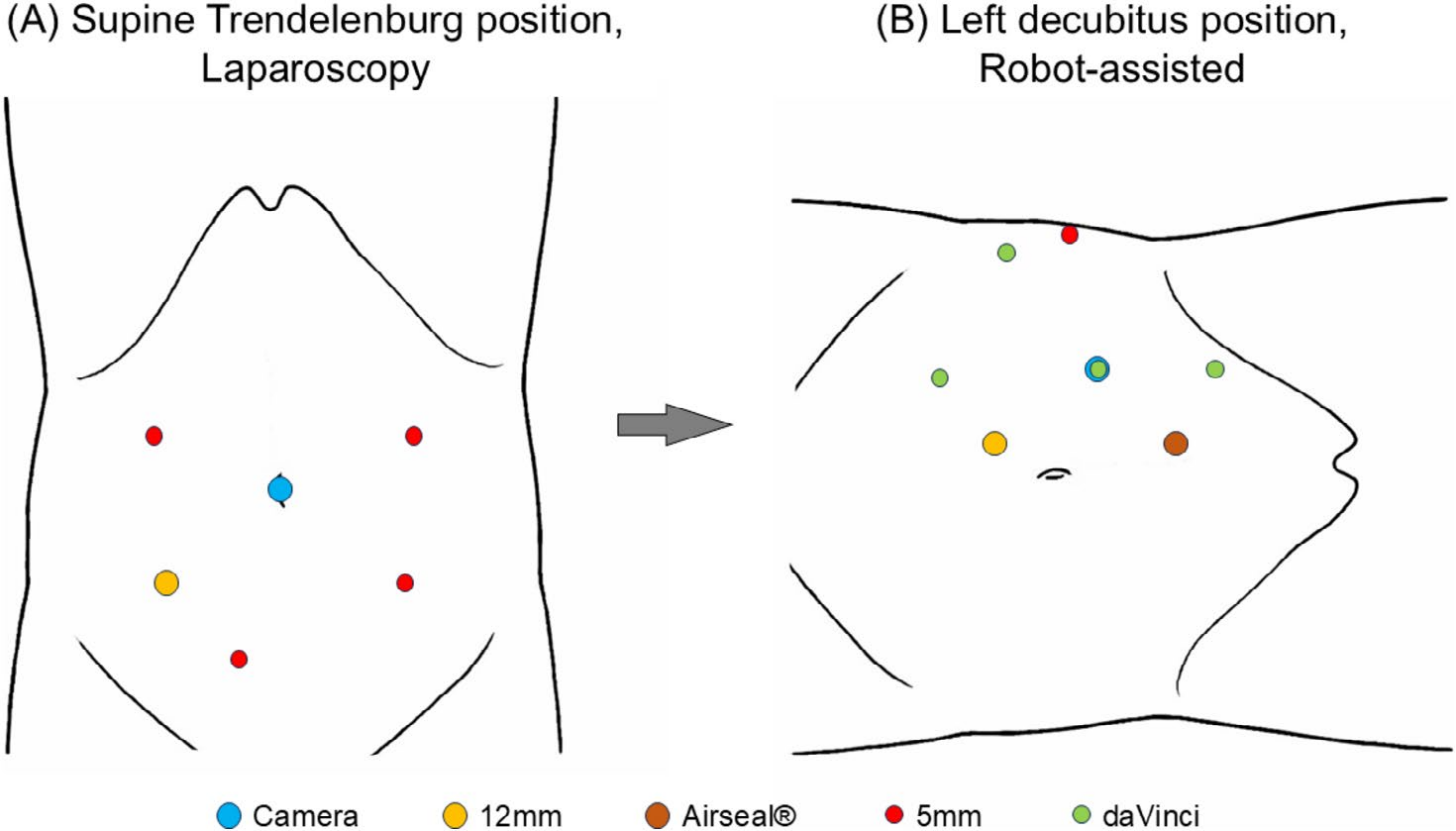
Port placement during surgery. (A) Approach in the supine, Trendelenburg position for management of the renal artery located posterior to the isthmus. Ports were arranged concentrically around the umbilicus for laparoscopic manipulation. (B) Standard port placement for robot‐assisted nephroureterectomy after control of the target renal artery. The patient was repositioned to the left lateral decubitus position.

**FIGURE 3:**
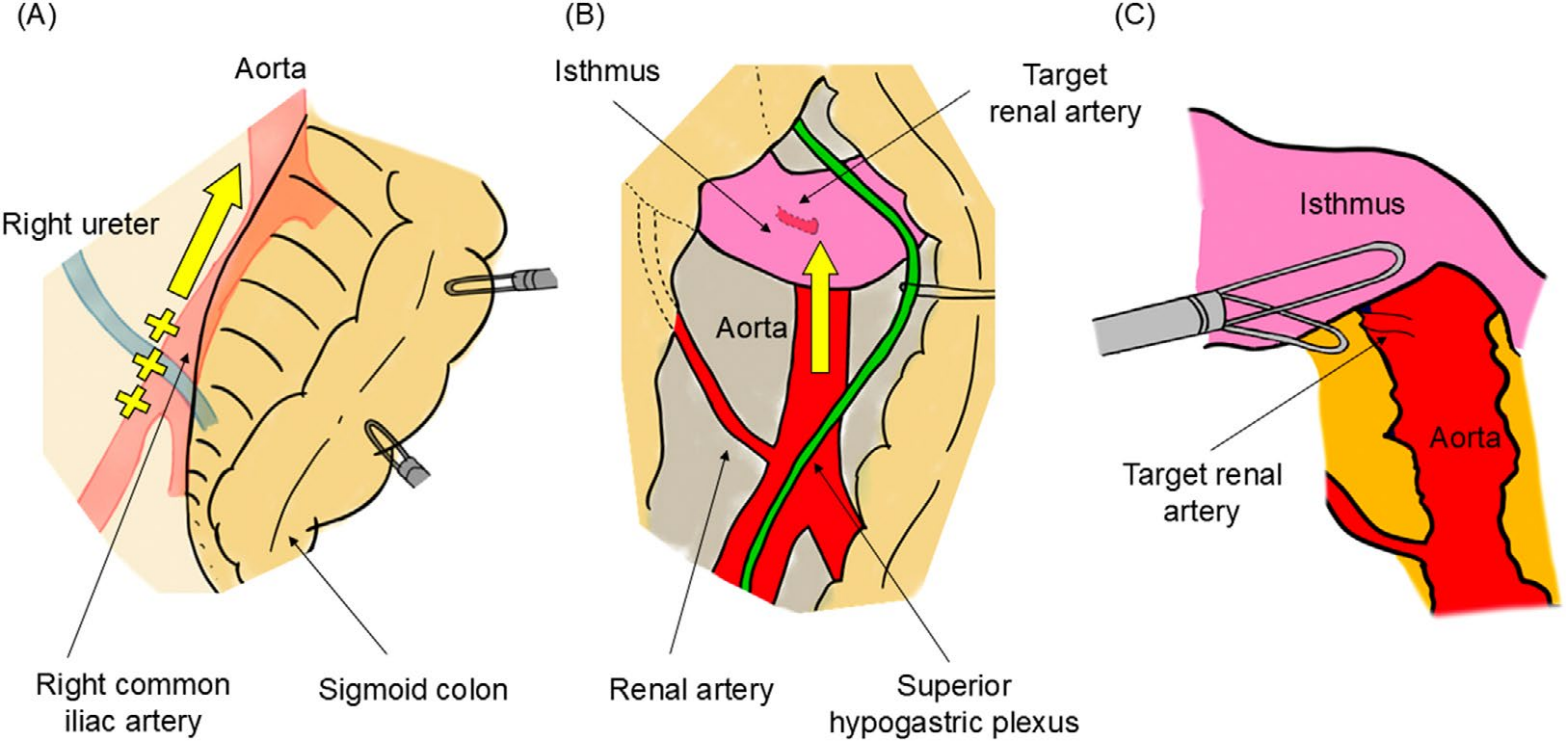
Surgical schema. (A) The peritoneum was incised internal to the sigmoid colon at the site indicated by the yellow cross, and dissection was advanced between the sigmoid mesocolon and retroperitoneal space in the direction indicated by the yellow arrow. (B) Dissection was continued along the anterior surface of the aorta using the superior hypogastric plexus as a landmark, as indicated by the yellow arrow. (C) The isthmus of the horseshoe kidney and the target renal artery located posterior to the isthmus were identified.

**FIGURE 4:**
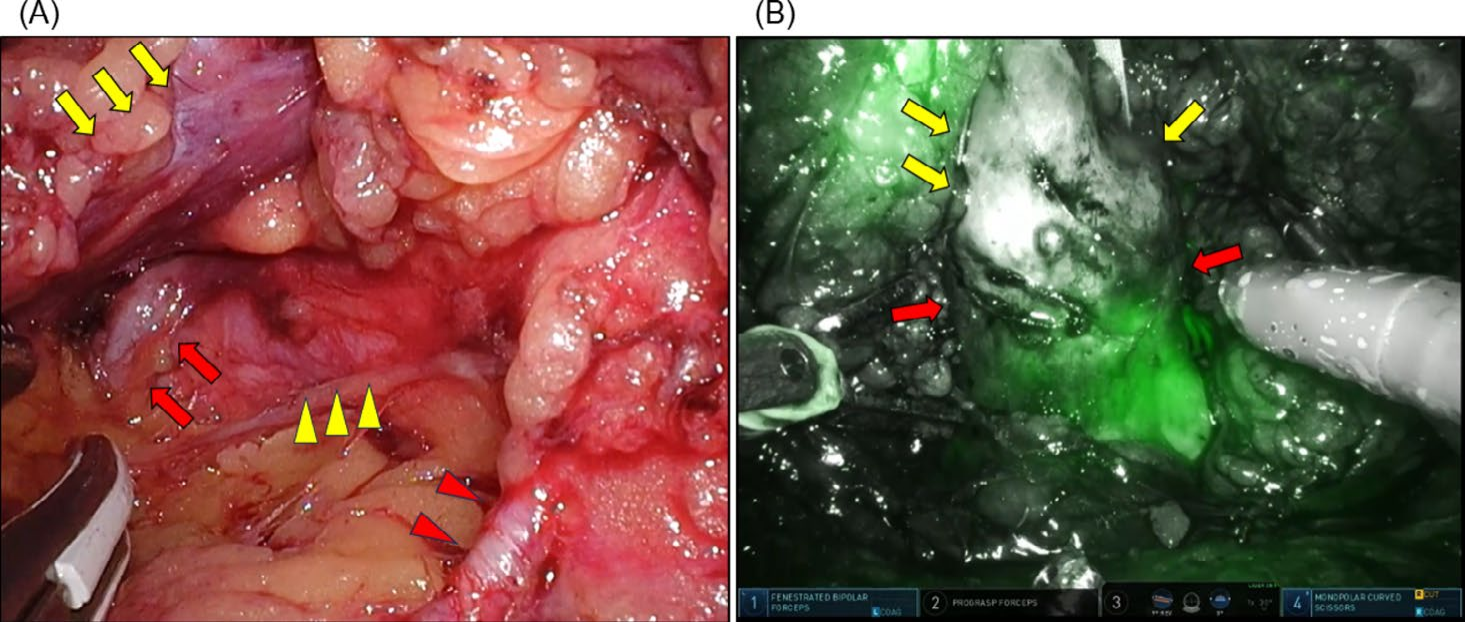
Intraoperative findings. (A) Red arrows indicate the renal artery located posterior to the isthmus; yellow arrows indicate the isthmus; red arrowheads indicate the caudal renal artery directed toward the lower pole; and yellow arrowheads indicate the aorta. (B) Red arrows indicate the boundary of the fused isthmus, clearly delineated by ICG; yellow arrows indicate the ischemic right side of the horseshoe kidney.

As this short renal artery was anticipated to be difficult to manage in the standard left lateral decubitus position, the procedure was initiated laparoscopically in the supine Trendelenburg position (Figure 2A). Because of limitations in the size and layout of our operating room, appropriate robotic roll‐in and docking from the required direction were not feasible during this phase. The peritoneum internal to the sigmoid colon was incised, and dissection was initiated between the sigmoid mesocolon and retroperitoneum in a medial‐to‐lateral fashion, as used in sigmoid colon and rectal cancer surgery [8] (Figure 3A). The superior hypogastric plexus was preserved as a landmark, and the dissection was extended cranially along the anterior surface of the aorta (Figure 3B). This approach allowed clear exposure of the isthmus and the renal artery running posterior to it, which was subsequently ligated using Hem‐o‐lok clips (Figures 3C and 4A).

The patient was then repositioned to the left lateral decubitus position, and robot‐assisted nephroureterectomy was continued in a standard manner (Figure 2B). Before dividing the isthmus, ICG (2 mL, 5 mg/mL) was intravenously administered to delineate the ischemic demarcation line (Figure 4B). The isthmus was divided using robotic monopolar scissors, and the cut surface was managed with soft coagulation and TachoSil. The operative time was 9 h 31 min, and blood loss was 270 mL. The patient recovered without any postoperative complications and was discharged on postoperative day 13.

## 3 Discussion

Standard right nephroureterectomy is typically performed in the left lateral decubitus position after bowel mobilization. In this case, preoperative imaging showed that one of the four renal arteries originated from the aorta posterior to the isthmus, entered its dorsal aspect, and was relatively short. Therefore, vascular access and control using the conventional approach were expected to be difficult. A medial approach, similar to colorectal surgical techniques used for sigmoid colon and rectal cancer [8], was adopted. This strategy allowed clear identification of the isthmus and renal artery and safe vascular control.

Horseshoe kidneys exhibit substantial vascular variability [3]. Horseshoe kidneys have approximately twice as many renal arteries as normal kidneys, with a mean of 4.57 arteries, and that approximately 40% originate distal to the inferior mesenteric artery [9]. In this case, four renal arteries supplied the right kidney, and three of these arteries arose caudal to the inferior mesenteric artery. However, to our knowledge, no previous reports specifically described technical difficulty in vascular control requiring modification of surgical approach in urological oncologic procedures. All reported cases of robot‐assisted surgery for upper tract urothelial carcinoma in a horseshoe kidney were performed using conventional patient positioning and a transperitoneal approach [4, 5, 6, 7]. Therefore, given the high frequency of vascular anomalies, the present approach may be useful in cases involving arteries located posterior to the isthmus.

Preservation of the superior hypogastric plexus is important in this approach. This plexus originates from the lumbar splanchnic nerves and the aortic plexus and is typically located at the L4–L5 level [10]. A review of surgical cases of colorectal cancer in patients with a horseshoe kidney revealed that the superior hypogastric plexus most commonly runs ventral to the isthmus, although dorsal courses have also been observed [8]. In this case, the plexus was located ventral to the isthmus. Given a previous report of retrograde ejaculation following laparoscopic nephrectomy in patients with a horseshoe kidney [11], careful identification and preservation of this plexus is essential.

Additionally, accurate identification of the isthmus transection line is critical to ensure safe division. In this case, ICG fluorescence imaging was used to confirm the transection line, as previously reported [7, 12]. Although the anatomical boundary could be recognized under white light, ICG fluorescence imaging helped delineate the perfusion border and ischemic area, thereby potentially reducing bleeding during isthmus division. This may be particularly beneficial when asymmetric isthmus fusion obscures the anatomical boundary.

Controlling bleeding during isthmus division remains a key issue. Several reports have described the use of staplers for isthmus division [13, 14]. Sozener et al. suggested that stapling is feasible when the isthmus is < 7 cm wide and 2 cm thick [13]. We did not use a stapler because the posterior aspect of the isthmus could not be sufficiently visualized during the left lateral decubitus position, and the divided vascular stump remained behind the isthmus, raising concern for inadvertent vascular injury. Instead, monopolar scissors allowed for isthmus division with minimal blood loss. This suggests that monopolar division may be a safe and effective alternative when feeding vessels are adequately controlled and the transection line is clearly identified, including with ICG use.

The prolonged operative time was likely due to complex vascular anatomy, repositioning, careful vascular control, and obesity‐related technical difficulty (BMI, 29 kg/m^2^). Thus, the benefit of this approach for safe vascular control should be balanced against the possibility of longer operative time.

Overall, this case illustrates a tailored surgical strategy in which patient positioning, vascular control, ICG‐guided transection line confirmation, and the method of isthmus division were selected according to the anatomical and vascular features.

## 4 Conclusion

A renal artery located posterior to the isthmus was safely managed using a medial approach with cranial dissection along the aorta. This case highlights the importance of a detailed preoperative vascular assessment and tailored surgical planning in horseshoe kidneys.

## Ethics Statement

The authors have nothing to report.

## Consent

Informed consent was obtained from the patient, and confidentiality was ensured.

## Conflicts of Interest

The authors declare no conflicts of interest.

## Data Availability

The data that support the findings of this study are available on request from the corresponding author. The data are not publicly available due to privacy or ethical restrictions.
